# Acute Epiglottitis with Concurrent Pneumonia and Septic Shock in an Alcoholic Adult Patient

**DOI:** 10.5811/cpcem.2018.9.39280

**Published:** 2018-10-16

**Authors:** Alexandra Chitty, Kevin Taylor

**Affiliations:** St. Lucie Medical Center, Department of Emergency Medicine, Port St. Lucie, Florida

## Abstract

Historically epiglottitis has been considered a childhood disease. However, the introduction of the *Haemophilus influenzae* type B vaccine has decreased the incidence of epiglottitis in children. It is important to recognize modern epiglottitis as a disease of adults. This report describes a case of acute bacterial epiglottitis in an adult patient secondary to infection caused by *Streptococcus pyogenes*, a group A streptococcal infection. This case demonstrates the importance of early recognition of epiglottitis in adults, as they can experience rapid clinical decline. The progression of this disease can lead to abrupt airway obstruction necessitating emergent airway management.

## INTRODUCTION

Epiglottitis is a rare and potentially life-threatening disease that leads to edema and inflammation of the supraglottic tissues, which may include the epiglottis, aryepiglottic folds, arytenoids, and adjacent tissue.^1^ After the introduction of the *Haemophilus influenzae* type B vaccine in 1988, the incidence of epiglottitis in children decreased.^1^,^3^ In 2006, there were 1.6 cases of epiglottitis per 100,000 adult patients (≥ 20 years old) and 0.5 cases per 100,000 pediatric patients (< 20 years old).^1^ Epiglottitis is traditionally thought to be caused by an infectious etiology, but it is important to consider other potential etiologies such as thermal insult, caustic insult, and trauma from foreign body ingestion.^1^

The emergency physician (EP) must have a high clinical index of suspicion for epiglottitis, as securing the airway early in the course of disease can prevent devastating complications.^1^ The surface epithelium of the epiglottis is highly vascularized and contains many lymphatic vessels making the area prone to rapid spread of infection and resultant inflammation.^1^ We present here a novel case of acute bacterial epiglottitis caused by a less-frequent organism and complicated by concurrent pneumonia in an alcoholic adult patient. The case also outlines the patient’s rapid clinical decline requiring establishment of an emergent airway in the emergency department (ED).

## CASE REPORT

A 58-year-old female smoker with a history of alcohol abuse presented to the ED at a community hospital with a chief complaint of shortness of breath, sore throat, and fever. She reported progressively worsening sore throat and odynophagia over the preceding two days as well as voice change and shortness of breath on the day of presentation to the ED. The patient admitted to fever, chills, diaphoresis, cough productive of phlegm, pain in the sides of her neck, and nausea. She denied sick contacts or recent travel.

On arrival to the ED, the patient’s vital signs were temperature 38.3° Celsius; pulse rate 130 beats per minute; blood pressure 118/72 millimeters of mercury; respiratory rate 20 breaths per minute; and pulse oximetry 99% on room air. The patient was acutely ill appearing with diaphoresis. She was able to phonate in complete sentences. She had full, active range of motion of her neck. On examination of the posterior oropharynx, there was no appreciable edema or peritonsillar abscess noted. She did have a small amount of frothy yellow phlegm in her posterior oropharynx and appeared unable to swallow these secretions. Auscultation of her lungs demonstrated diffuse rhonchi and slight expiratory wheeze.

Sepsis was suspected on arrival, and the ED sepsis protocol was initiated with a suspected source of community-acquired pneumonia. The sepsis protocol included administration of intravenous (IV) fluids at 30 milliliters (mL) per kilogram of body weight and broad-spectrum antibiotics. The patient was given two grams of IV ceftriaxone and 500 milligrams (mg) of IV azithromycin as well as 10 mg of IV dexamethasone. We ordered an albuterol and ipratropium nebulizer treatment as the patient was suspected to have undiagnosed, underlying chronic obstructive pulmonary disease given her smoking status and lung examination.

One hour after initial ED evaluation, the patient was receiving the nebulized breathing treatment when she was found to be sitting up on the side of her stretcher in acute respiratory distress with an oxygen saturation in the seventies. She was anxious appearing and unable to phonate. The patient was immediately transferred to a nearby resuscitation bay. She was placed on 15 L of oxygen by nasal cannula as well as on a non-rebreather mask. Materials for an emergent surgical airway were readied at bedside.

Staff included two nurses, a respiratory therapist, an emergency medicine (EM) attending physician, and two EM resident physicians. Rapid sequence intubation was initially attempted by the EM resident using video laryngoscopy. Upon visualization, we noted significant edema and purulence involving the epiglottis, arytenoids, and adjacent soft tissues causing severe deformity of normal anatomy, which obscured visualization of the glottis. There was pooling of secretions in the supraglottic region as well. The supraglottic tissues were bulbous and friable with diffuse exudates and active bleeding. While initial intubation attempts using the video laryngoscope and a bougie were unsuccessful, the attending physician was able to successfully secure the airway with an endotracheal tube placed with over-the-bougie technique. Fortunately, we were able to achieve adequate ventilation via bag-valve-mask between direct laryngoscopy attempts preventing need for a surgical airway.

Upon review of the laboratory results, the patient was noted to have a white blood cell count of 18,300 cells per microliter and an initial point of care lactic acid of 5.51 millimoles per liter (of note, value was obtained prior to treatment with albuterol). Group A streptococcal testing of the oropharynx was positive. Her troponin I level was elevated at 0.068 nanograms per mL and felt to be secondary to sepsis. Computed tomography (CT) of the chest with IV contrast demonstrated bilateral patchy consolidative changes in the lower lobes consistent with pneumonia, a thickened distal esophagus with retained fluid, multiple wedge-shaped hypodensities in the kidneys consistent with renal infarcts, and hepatic steatosis. A CT of the neck with IV contrast demonstrated enlargement of the tonsils with adjacent edema and narrowing of the airway without definite fluid collection. Following intubation the patient became hypotensive, necessitating placement of a central venous catheter and the use of norepinephrine for continued management of septic shock. The patient was admitted to the intensive care unit.

**CPC-EM Capsule**

What do we already know about this clinical entity?

Acute epiglottitis is a rare but serious condition that can result in life-threatening airway obstruction.

What makes this presentation of disease reportable?

This is the first presentation reported in the literature of acute epiglottitis in an adult with concurrent pneumonia caused by Streptococcus pyogenes.

What is the major learning point?

We highlight the importance of early assessment and planning in patients with potential threats to airway patency.

How might this improve emergency medicine practice?

This case demonstrates the importance of having an increased awareness of this disease, especially among adult patients, as the consequences of unrecognized acute epiglottitis can be fatal.

The patient was extubated the following day, and she was transferred to another facility two days following admission for otolaryngology evaluation. Upon arrival to the second hospital, the patient was evaluated by an otolaryngologist who performed flexible laryngoscopy, which demonstrated epiglottitis with a mottled epiglottis and fibrinous exudate that appeared to be consistent with early resolution of epiglottitis. The patient was continued on IV steroids as well as IV broad-spectrum antibiotics. She continued to require oxygen supplementation to maintain an oxygen saturation above 88%. Respiratory cultures and blood cultures were positive for *Streptococcus pyogenes*. The patient was discharged from the hospital 15 days following her initial presentation to the ED, the completion of the course of IV antibiotics and the resolution of hypoxia.

## DISCUSSION

Clinical suspicion and early recognition of acute epiglottitis is critical in the ED as this disease can progress rapidly and lead to sudden airway obstruction.^1^ Because direct visualization of the epiglottis is not always possible on initial presentation, it is important to recognize clinical features of epiglottitis, which may include sore throat, fever, stridor, drooling, dysphagia, odynophagia, pooling of secretions in the oropharynx, “tripoding,” anxiety, respiratory distress, and muffled voice.^1^ In addition to recognizing the clinical features of epiglottitis, EPs should be aware of certain risk factors that increase an adult patient’s risk of developing epiglottitis. Notable risk factors for epiglottitis in adult patients include hypertension, diabetes mellitus, substance abuse, and immune deficiency.^1^ Some factors associated with an increase in severity of epiglottitis include body mass index greater than 25, diabetes mellitus, concurrent pneumonia, and the presence of an epiglottic cyst at the time of admission.^4^

While the focus of this case report was to highlight the importance of early recognition of epiglottitis, it is important to note that certain studies and interventions should be initiated promptly. One may consider ordering a lateral neck radiograph if the patient appears stable.^1^ Width of the epiglottis greater than eight millimeters and aryepiglottic fold width greater than seven millimeters measured on lateral neck radiograph appears be diagnostic of acute epiglottitis in an adult patient.^5^ However, diagnosis is typically confirmed by direct visualization of the epiglottis.^1^ Broad-spectrum antibiotics and IV hydration are crucial measures in the treatment of this disease.^6^ The first-line recommended antibiotic regimen for acute epiglottitis is cefotaxime 50 mg per kilogram body weight intravenously every eight hours plus vancomycin 15 mg per kilogram body weight intravenously every 12 hours.^6^ The use of steroids may be considered to help decrease inflammation and edema of the airway.^6^

Upon review of this case, the patient’s alcohol abuse was her most significant risk factor for epiglottitis, and it is important to be cognizant of the immunosuppression caused by this manner of substance abuse.^7^ Chronic alcohol use can accelerate inflammatory responses and increase the susceptibility of patients to viral and bacterial infections as well as to sterile inflammation.^7^ This patient also had multiple factors that increased her likelihood of a severe episode of epiglottitis including a body mass index of 26.1 and concurrent pneumonia.

## CONCLUSION

This case demonstrates the importance of early identification of clinical signs and symptoms as well as risk factors for epiglottitis. Adult patients with epiglottitis can rapidly develop airway compromise. By early identification of epiglottitis, the EP may be able to adequately prepare, manage, and prevent progression to surgical airway. Additionally, this case underscores the importance of evaluating high-risk patients for other sources of infection including pneumonia as these may be contributory and require additional treatment measures. The patient presented here ultimately achieved a good outcome and no surgical airway intervention was required. However, this case could have easily had a negative or adverse outcome, which is why we emphasize the importance of maintaining a high clinical index of suspicion for epiglottitis based on appropriate clinical presentation. Frequent reassessments, avoidance of anchoring bias, and early recognition of airway compromise are crucial.

Documented patient informed consent and/or Institutional Review Board approval has been obtained and filed for publication of this case report.
